# Sex-Dependent Effects of Cardiometabolic Health and *APOE4* on Brain Age: A Longitudinal Cohort Study

**DOI:** 10.1212/WNL.0000000000209744

**Published:** 2024-08-22

**Authors:** Sivaniya Subramaniapillai, Louise S. Schindler, Paul Redmond, Mark E. Bastin, Joanna M. Wardlaw, Maria Valdés Hernández, Susana Muñoz Maniega, Benjamin Aribisala, Lars T. Westlye, William Coath, James Groves, David M. Cash, Josephine Barnes, Sarah-Naomi James, Carole H. Sudre, Frederik Barkhof, Marcus Richards, Janie Corley, Tom C. Russ, Simon R. Cox, Jonathan M. Schott, James H. Cole, Ann-Marie G. de Lange

**Affiliations:** From the Department of Clinical Neuroscience (S.S., L.S.S., A.-M.G.d.L.), Lausanne University Hospital and University of Lausanne, Switzerland; Department of Psychology (P.R., M.E.B., J.M.W., M.V.H., S.M.M., B.A., J.C., T.C.R., S.R.C.), University of Edinburgh, United Kingdom; Department of Psychology (L.T.W.), University of Oslo, Norway; Dementia Research Centre (W.C., J.G., D.M.C., J.B., S.-N.J., C.H.S., J.M.S.), Centre for Medical Image Computing (C.H.S., F.B., J.H.C.), and MRC Unit for Lifelong Health and Ageing (M.R., S.-N.J., C.H.S.), University College London, United Kingdom.

## Abstract

**Background and Objectives:**

The aging population is growing faster than all other demographic strata. With older age comes a greater risk of health conditions such as obesity and high blood pressure (BP). These cardiometabolic risk factors (CMRs) exhibit prominent sex differences in midlife and aging, yet their influence on brain health in females vs males is largely unexplored. In this study, we investigated sex differences in relationships between BP, body mass index (BMI), and brain age over time and tested for interactions with *APOE* ε4 genotype (*APOE4*), a known genetic risk factor of Alzheimer disease.

**Methods:**

The sample included participants from 2 United Kingdom–based longitudinal birth cohorts, the Lothian Birth Cohort (1936) and Insight 46 (1946). Participants with MRI data from at least 1 time point were included to evaluate sex differences in associations between CMRs and brain age. The open-access software package brainageR 2.1 was used to estimate brain age for each participant. Linear mixed-effects models were used to assess the relationships between brain age, BMI, BP, and *APOE4* status (i.e., carrier vs noncarrier) in males and females over time.

**Results:**

The combined sample comprised 1,120 participants (48% female) with a mean age (SD) of 73 (0.72) years in the Lothian Birth Cohort and 71 (0.68) years in Insight 46 at the time point 1 assessment. Approximately 30% of participants were *APOE4* carriers. Higher systolic and diastolic BP was significantly associated with older brain age in females only (β = 0.43–0.56, *p* < 0.05). Among males, higher BMI was associated with older brain age across time points and *APOE4* groups (β = 0.72–0.77, *p* < 0.05). In females, higher BMI was linked to older brain age among *APOE4* noncarriers (β = 0.68–0.99, *p* < 0.05), whereas higher BMI was linked to younger brain age among carriers, particularly at the last time point (β = −1.75, *p* < 0.05).

**Discussion:**

This study indicates sex-dependent and time-dependent relationships between CMRs, *APOE4* status, and brain age. Our findings highlight the necessity of sex-stratified analyses to elucidate the role of CMRs in individual aging trajectories, providing a basis for developing personalized preventive interventions.

## Introduction

With the global aging population on the rise, addressing the prevalence of cardiometabolic risk factors (CMRs) such as hypertension and obesity has become paramount. These risk factors carry significant implications for health outcomes in older adults, including an elevated risk of cardiometabolic diseases,^1^ accelerated brain aging,^2^ and Alzheimer disease (AD).^3^

Recent cross-sectional studies indicate prominent sex differences in the impact of both cardiometabolic and genetic risk factors including *APOE* ε4 (*APOE4*+) on brain health.^4,5^ However, the dynamics of these influences over time remain largely unexplored. By examining the interplay between CMRs and *APOE* genotype at different time points in older adulthood, we can better understand sex-specific risk profiles associated with brain health in aging.

Previous studies have demonstrated that prediction of the brain's biological age provides a sensitive neuroimaging-based marker for brain health and disease.^2,6-9^ Brain age gap (BAG) represents the difference between an individual's chronological age and their predicted brain age derived from structural brain characteristics. Negative BAG values indicate a “younger” brain age relative to chronological age and are associated with positive health outcomes, such as better physical health and cognitive function.^10,11^ Conversely, positive BAG values indicating an “older” brain age may reflect the rate of biological aging, with increasing values over time potentially reflecting accelerated brain deterioration and aging.^12^ Positive BAG values have been associated with cognitive impairments, mortality, and elevated cardiometabolic and neurodegenerative risk.^7,8,11,12^

Among the most common CMRs are markers of obesity and high blood pressure (BP), both of which have been linked to brain morphological differences.^13^ While elevated BP has been consistently associated with steeper rates of brain aging^14^ and older brain age^2,6,8^ for both males and females, the associations between brain health and body fat exhibit a more complex pattern across the life course.^15^ For instance, a large-scale study showed that body mass index (BMI) measured more than 20 years before a dementia diagnosis was positively correlated with dementia risk, whereas BMI measured less than 10 years before diagnosis was negatively correlated with dementia risk.^15^ Thus, although higher BMI in midlife might predominantly reflect obesity, higher BMI in older age may reflect overall physical fitness or the absence of degenerative diseases.

The relationship between body composition and brain health may also vary between males and females across different life phases.^11^ For example, we previously showed in the UK Biobank study (n > 21,000) that greater BMI, waist-to-hip ratio (WHR), and body fat percentage (BF%) were consistently linked to older brain age in males across midlife and older adulthood.^4^ In females, however, greater WHR, but not BMI and BF%, was associated with older brain age. These differential effects were most prominent in the group of oldest females. Given that body fat serves as the primary source of estrogen in postmenopausal females, higher levels may potentially offer protection against neurodegenerative processes.^16^ However, low BMI could also indicate signs of frailty, sarcopenia, or preclinical dementia in later life stages,^5,17^ which could be reflected in the group of oldest females from our previous study.

In addition to sex differences in associations between CMRs and brain health across different life phases, the risk of neurodegeneration conferred by the *APOE4*+ genotype is also known to differ between males and females^5,18^ and may interact with markers of cardiometabolic health.^19^ For example, in our previous work using the North American PREVENT-AD cohort of cognitively normal participants, we observed that the presence of a family history of AD and the *APOE4*+ genetic risk was associated with older brain age in females than in males with similar risk levels. In females, higher BMI was associated with younger brain age, with stronger associations observed among those with identified AD risk factors.^5^ While these studies provide evidence of sex differences in the role of CMRs and *APOE* genotype in brain health and aging, the cross-sectional data limit our understanding of how these patterns may change with increasing age. Hence, longitudinal investigations could help to identify risk profiles for adverse brain health and clarify critical age windows where CMRs may exert sex-specific and genotype-specific effects on the brain.

In this study, we used longitudinal data from the Lothian Birth Cohort 1936 (LBC1936)^20,21^ and Insight 46^22^ to investigate the impact of key CMRs on brain age throughout different stages of the aging process in males and females. These cohorts consist of participants from the United Kingdom who were born within the same year (1936 and 1946, respectively), minimizing potential variations that could be attributed to differences in age. We applied a previously established brain age model^7,23,24^ to predict participants' brain age across the 3 time points in older adulthood. Subsequently, we used linear mixed-effects regression models to examine the relationships between BAG, BMI, systolic/diastolic BP, and *APOE4* status in males and females over time.

## Methods

### Sample Characteristics

#### Lothian Birth Cohort 1936

The study cohort comprised participants from the LBC1936, a community-based sample from Edinburgh and the Lothians, Scotland.^20,21,25^ Participants were all born in 1936 and were selected from the Scottish Mental Survey of 1947, which aimed to test the intelligence of 70,805 children attending school in Scotland in June 1947. This sample was assessed in older adulthood for cognitive, medical, physical, biological, and lifestyle factors. Between 2004 and 2007, participants from this larger survey cohort were recruited to wave 1 of the LBC1936 study, with an average age of 70 years. Subsequent waves occurred in 2007–10 (wave 2), 2011–13 (wave 3), and 2014–17 (wave 4). On average, there were 3 years between waves. The inclusion criteria required completion of the Scottish Mental Survey in 1947 and the absence of neurodegenerative diseases at wave 2. T1-weighted MRI scans were acquired on the same 1.5T using a GE Signa Horizon HDxt clinical scanner. Comprehensive details on cohort collection are available in the specified articles by Deary et al.^20,25^

#### Insight 46

The Medical Research Council National Survey of Health and Development is a birth cohort study, which initially followed 5,362 individuals from Britain since their birth in March 1946.^26^ A random subsample of participants aged 69–71 years joined a neuroscience substudy called Insight 46, in which they underwent assessments including clinical and cognitive tests and simultaneous MRI and ^18^F-florbetapir PET imaging. The selection criteria involved random sampling from participants aged 60–64 years who had previously expressed a willingness to attend a clinic visit in London and for whom relevant childhood and adulthood data were available. All assessments took place at a single site, with recruitment occurring in 2015 (time point 1), when participants were around 69 years old. A follow-up assessment took place approximately 24 months later in 2016, with the scan interval ranging from 2 to 4.5 years.

In both cohorts, the inclusion criteria included the availability of at least 1 T1-weighted MRI scan, which passed quality control. There were no selection criteria for participants at subsequent MRI time points because the purpose of the study was to investigate aging trajectories over time without any constraints. T1-weighted MRI scans were acquired using a 3T Siemens Biograph mMR combined PET/MRI scanner (Siemens Healthcare, Erlangen, Germany). More information on the Insight 46 study design and recruitment can be found in studies by Lane et al. and Mason et al.^22,27^

### Standard Protocol Approvals, Registrations, and Patient Consents

All participants in both cohorts provided informed written consent. The LBC1936 study received ethical approval from the Multicentre Research Ethics Committee for Scotland (MREC/01/0/56), the Lothian Research Ethics Committee (LREC/2003/2/29), and the Scotland Research Ethics Committee (07/MRE00/58). The Insight 46 study received ethical approval from the National Research Ethics Service Committee London (14/LO/1173).

### MRI Data Preparation and Brain Age Prediction

The brain age of each participant was estimated using brainageR 2.1,^24^ an open-source software program that generates brain-predicted age from raw T1-weighted MRI scans. The brainageR model was previously trained using a Gaussian Process Regression to predict age from brain volumetric maps of 3,377 healthy individuals (mean age = 40.6 years, SD = 21.4, age range 18–92 years) across 1.5T and 3T scans from 7 publicly available data sets. The trained model was then tested on 857 holdout participants (mean age = 40.1 years, SD = 21.8, age range 18–90 years) originating from the 7 data sets with model performance as follows: Pearson correlation between chronological age and brain-predicted age: r = 0.97, mean absolute error = 3.93 years, and R^2^ = 0.95. The model was also tested using an independent data set, CamCAN, which included 611 participants aged 18–90 years with model performance demonstrating r = 0.95 and mean absolute error = 4.90 years. Thus, brainageR has demonstrated high prediction accuracy through internal and external validation and across different scanner strengths.^7,23,24^ The LBC1936 and Insight 46 data were not used in the training or validation of brainageR. The rotation matrix from the pretrained model was applied to the new imaging data to predict age in our sample.

Before prediction, the images were segmented and normalized with SPM12 software. During this preprocessing stage, the FSL *slicesdir* function^28^ generated 2-dimensional slices of the segmentation and normalization outputs for quality control. Subsequently, visual quality control was performed resulting in the removal of n = 7 images from the LBC1936 due to motion artifacts. The normalized images were then converted to gray matter, white matter, and CSF vectors. These vectors were masked using 0.3 threshold based on the mean image template from the brainageR training data set and then concatenated.^7^

Next, the brainageR model was applied to the masked study images to predict age. For each image, brain-predicted age with 95% CIs was calculated, and the BAG was obtained by subtracting chronological age from brain-predicted age.

### Cardiometabolic Risk Factors

The CMRs were BMI, systolic BP, and diastolic BP. These measures were chosen because of their consistent availability and measurement methods in both data sets, enabling the reliable combination of samples to attain a larger sample. Previous research has found sex differences in the levels of BMI and BP,^29^ further justifying our exploration into their influence on brain health in this study. Participants with BMI ≥40 (n = 36) were excluded because these values may reflect morbid obesity and risk of serious health comorbidities.^30^ BP was measured while participants were sitting, standing, or lying down. For Insight 46 participants, we used the average of 2 BP measurements that were taken at the same time point while participants were lying down. For LBC1936 participants, although multiple BP readings were taken during each appointment, we used the first available BP measurement while participants were sitting down to maximize the use of available data from this cohort. The correlations between BMI, systolic BP, and diastolic BP for the combined sample are displayed in eFigure 1.

### APOE Genotyping

*APOE4* carrier status was identified by assessing 2 *APOE* single-nucleotide variants (rs7412 and rs429358) through the application of TaqMan technology.^22^ *APOE4* status was classified as “carrier” for the combinations of ε2/ε4, ε3/ε4, and ε4/ε4 and “noncarrier” for the combinations of ε2/ε2, ε2/ε3, and ε3/ε3. The percentage of ε4 carriers remained relatively consistent at approximately 30% across time points.

### Statistical Analyses

The statistical analyses were performed using R version 3.6.2. We ran linear mixed-effects regressions (*lmer*)^31^ with participant ID as a random intercept to test for (1) main effects of sex, time point, *APOE4* status, and each CMR individually on BAG and (2) sex differences in associations between CMRs, *APOE4* status, time point, and BAG. Post hoc pairwise comparisons were conducted using the “emmeans” package in R.^32^ We conducted the main analyses by combining both data sets, to increase sample size and capture information on the third time point available from the LBC1936.

The CMRs (BMI, systolic BP, and diastolic BP) were standardized by subtracting the mean and dividing by the standard deviation across all available measures from the combined data at each time point. Sex (2: male, female), *APOE4* status (2: carrier, noncarrier), time point (3: time point 1, time point 2, time point 3), and data set (2: LBC1936, Insight 46) were treated as categorical variables in the primary analyses. We used time point as a categorical variable to capture and compare differences in associations between age periods, simplifying the interpretation of the results—particularly for the complex 3-way interaction models. To account for the variations in participants' ages within and across time points across data sets, we included the age of participants at each time point as a covariate in our models.

To account for multiple comparisons, we adjusted the *p*-values using false discovery rate correction according to the Benjamini-Hochberg method with a significance threshold set at 0.05.^33^ We used F-tests to interpret the models including categorical variables with more than 2 levels. We calculated the *F*-statistics using the *anova* wrapper function with the type set to “III” to compute type-III sum of squares for each of the *lmer* models. While CMRs and brain health measures may exhibit nonlinear relationships over the adult life course, we tested for linear relationships between BAG and the independent variables, given the narrow age range of the participant cohorts.^34^ Finally, we used the “effects” package in R to visualize findings obtained from the models.^35^

We first tested for the main effects of sex, time point, *APOE* genotype, BMI, systolic BP, and diastolic BP on BAG using the separate *lmer* models for each variable of interest:

$$\text{Model}\;1:\text{BAG}=\beta_{0}+\beta_{1}x+\beta_{2}\text{Age}+\beta_{3}\text{Dataset}+u+\epsilon$$

where BAG represents the BAG values from both males and females, x represents the variable of interest (sex, time point, *APOE* genotype, BMI, systolic BP, or diastolic BP), Age represents the participants' age at each time point, Dataset represents whether the participant was from the LBC1936 or Insight 46 data set, u represents the modelling of participant ID as a random intercept, and ϵ is the error term. The global intercept is denoted by $\beta_{0}$ while the regression coefficients for x, Age, and Dataset are denoted as $\beta_{1}$, $\beta_{2}$, and $\beta_{3}$, respectively.

We then tested whether there were sex differences in the effects of CMRs (BMI, systolic BP, diastolic BP) on BAG while accounting for the time point:

$$\text{Model}\;2:\text{BAG}=\beta_{0}+\beta_{1}\text{CMR}\times \text{Sex}+\beta_{2}\text{Timepoint}+\beta_{3}\text{Age}+\beta_{4}\text{Dataset}+u+\epsilon$$

We ran additional analyses to investigate the interaction between sex and *APOE4* status and sex and time point on BAG.

Because our primary goal was to investigate the association between sex and CMRs over time, leveraging the use of the longitudinal data, the following *lmer* models were used to test the 3-way interactions between sex, CMRs, and time point:

$$\text{Model}\;3:\text{BAG}=\beta_{0\;}+\beta_{1}\text{Sex}+\beta_{2}\text{CMR}+\beta_{3}\text{Timepoint}+\beta_{4}\text{CMR}\times \text{Sex}\times \text{Timepoint}+\beta_{5}\text{Age}+\beta_{6}\text{Dataset}+u+\epsilon$$

where $\beta_{4}$ represents the interaction terms of interest.

To derive the values for the associations between each CMR and BAG by time point within each sex, we performed the following post hoc *lmer* models in males and females separately:

$$\text{Model}\;4:\text{BAG}=\beta_{0\;}+\beta_{1}\text{CMR}+\beta_{2}\text{Timepoint}+\beta_{3}\text{CMR}\times \text{Timepoint}+\beta_{4}\text{Age}+\beta_{5}\text{Dataset}+u+\epsilon$$

We then tested whether there was an interaction effect of CMR and *APOE4* status with time point on BAG, using sex-specific subsamples of participants with genotype information available:

$$\text{Model}\;5:\text{BAG}=\beta_{0\;}+\beta_{1}\text{CMR}+\beta_{2}\text{APOE}+\beta_{3}\text{Timepoint}+\beta_{4}\text{CMR}\times \text{APOE}\times \text{Timepoint}+\beta_{5}\text{Age}+\beta_{6}\text{Dataset}+u+\epsilon$$

We report the main results from the analyses based on the combined cohorts in the Results section and present detailed post hoc analyses in eTables 3–16.

We conducted a series of supplementary analyses to ensure the consistency of our findings with the primary analyses (eMethods 1). These analyses involved excluding brain age outliers, conducting separate analyses within each data set, performing analyses after excluding the third time point from the LBC1936 cohort, and including only participants with data from 2 or more time points. We also conducted analyses accounting for covariates such as the presence of diabetes, alcohol intake, socioeconomic status, education level, and hormone therapy and excluding participants with dementia diagnosis and cognitive scores potentially indicative of mild cognitive impairment (eMethods 2).

### Data Availability

Access to Insight 46 data can be requested at skylark.ucl.ac.uk/NSHD/doku.php?id=home. To request access to LBC1936 data, visit lothian-birth-cohorts.ed.ac.uk/data-access-collaboration.

## Results

### Participants

The LBC1936 initially included 1,091 participants during wave 1. From this cohort, we selected 654 participants (mean age = 72.68 years, SD = 0.72) at wave 2 (referred to as time point 1 in our study) who had MRI data and relevant demographic information available. At wave 3, there were 471 participants (i.e., time point 2; mean age = 76.37 years, SD = 0.65), and there were 376 participants at wave 4 (i.e., time point 3; mean age = 79.44 years, SD = 0.65).

The Insight 46 included a sample of 502 participants collected at Phase 1. From this sample, 466 participants (i.e., time point 1; mean age = 70.53 years, SD = 0.63) at Phase 1 and 368 participants (i.e., time point 2; mean age = 72.48 years, SD = 0.59) at Phase 2 were eligible for our study. The combined sample comprised a total of 1,120 participants (48% female) at baseline. In both cohorts combined, the average number of years between time points is 3 years. Additional details on key demographic variables at time point 1 are presented in Table 1 and at subsequent time points in eTables 1 and 2 in the Supplement. eFigure 2 provides a flowchart of participant selection from the original birth cohorts.

**Table 1:** Sample Demographics at Time Point 1 for LBC1936 and Insight 46

	LBC1936 (n = 654)		Insight 46 (n = 466)		Combined (n = 1,120)	
	Male	Female	Male	Female	Male	Female
N (%)	347 (53)	307 (47)	240 (52)	226 (48)	587 (52)	533 (48)
Baseline age, y	72.63 (0.71)	72.73 (0.74)	70.66 (0.68)	70.67 (0.68)	71.82 (1.20)	71.86 (1.24)
*APOE4* status, n (%)						
ε4 carrier	96 (29)	86 (29)	77 (32)	63 (28)	173 (30)	149 (29)
ε4 noncarrier	235 (71)	207 (71)	161 (68)	163 (72)	396 (70)	370 (71)
ε2/ε2	1	1	0	0	1	1
ε2/ε3	42	35	33	33	75	68
ε2/ε4	7	6	6	5	13	11
ε4/ε3	192	171	128	130	320	301
ε3/ε4	82	74	66	51	148	125
ε4/ε4	7	6	5	7	12	13
*APOE4* status unavailable	16	14	2	6	18	14
Education, y	10.78 (1.17)	10.86 (1.11)				
No qualifications			24	31		
Vocational only			19	18		
O level or equivalent			40	56		
A level or equivalent			84	83		
Higher			73	38		
Education: combined^a^						
Low					246	205
Medium					224	257
High					117	71
MMSE	28.59 (1.58)	29.02 (1.15)	29.18 (1.03)	29.34 (0.91)	28.83 (1.41)	29.15 (1.07)
Social class/Townsend index	2.47 (1.00)	2.18 (0.82)	−1.12 (2.37)	−0.78 (3.18)		
SES: combined^a^						
Low					206	80
Medium					102	181
High					267	257
Unavailable					5	11
BMI, kg/m^2^	27.75 (3.65)	27.39 (4.13)	27.76 (3.68)	27.10 (4.57)	27.75 (3.66)	27.27 (4.32)
Systolic BP, mm Hg	153.25 (19.58)	151.15 (20.86)	139.84 (16.51)	136.29 (17.30)	147.78 (19.52)	144.85 (20.76)
Diastolic BP, mm Hg	80.26 (9.94)	78.73 (10.08)	74.20 (10.15)	73.98 (10.15)	77.79 (10.45)	76.72 (10.37)
Diabetes^a^	Yes: 46 No: 301	Yes: 19 No: 288	Yes: 23 No: 217	Yes: 19 No: 206	Yes: 69 No: 518	Yes: 38 No: 494
Stroke^a^	Yes: 22 No: 325	Yes: 24 No: 283	Yes: 14 No: 224	Yes: 6 No: 215	Yes: 36 No: 549	Yes: 30 No: 498
Cardiovascular disease^a^	Yes: 114 No: 233	Yes: 61 No: 246	Yes: 26 No: 208	Yes: 17 No: 207	Yes: 140 No: 441	Yes: 78 No: 453

Abbreviations: BMI = body mass index; BP = blood pressure; LBC1936 = Lothian Birth Cohort 1936; MMSE = Mini-Mental State Examination; SES = socioeconomic status.

Mean (SD) for key demographic variables.

a Refer to eMethod 2 for the calculation of education and SES combined, in addition to details on how additional cardiometabolic risk factors (diabetes, stroke, cardiovascular disease) were assessed.

### Main Effects of Sex, Time Point, and CMRs on BAG (Model 1)

**Figure 1:**
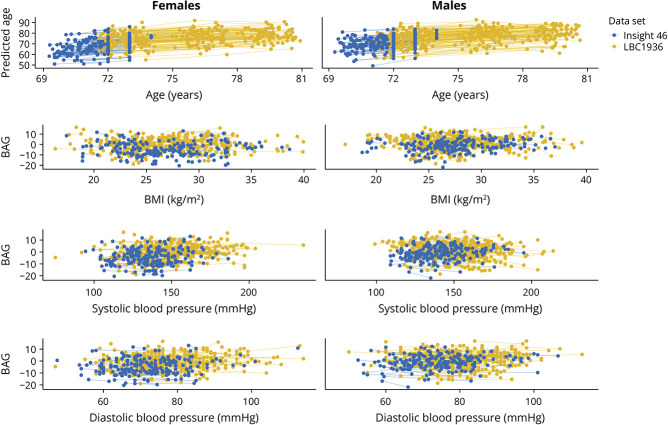
Relationships Between Predicted and Chronological Age and CMRs With BAG Longitudinal observations derived from a random sample of 50% of participants, depicting the relationships between participants' predicted and chronological age and CMRs with BAG, respectively. BAG = brain age gap; BMI = body mass index; BP = blood pressure; CMR = cardiometabolic risk factor.

In Figure 1, longitudinal data from a random sample of 50% of participants illustrates the relationship between participants' predicted and chronological age and CMRs with BAG, respectively, capturing all time points for each participants. Table 2 gives the main effects of sex, time point, and CMRs on BAG (model 1) for the combined data set. The results showed significant main effects of sex, revealing higher BAG values in males compared with females (eTable 3, eFigure 3a). Time point also showed a significant effect on BAG, with lower BAG values at time point 1 compared with later time points (eTable 3, eFigure 3b). Greater BMI, systolic BP, and diastolic BP were all significantly associated with higher BAG values (eTable 3).

**Table 2:** Main Effects of Sex, TP, BMI, Systolic BP, Diastolic BP, and *APOE4* Status on Brain Age Gap (Model 1)

Main effect	β	*F*	*p* Value	*p*corr
Sex	−2.31	39.87	3.88 × 10^−10^*	1.16 × 10^−9^*
TP		22.34	2.79 × 10^−10^*	1.16 × 10^−9^*
TP2 vs TP1	1.51			
TP3 vs TP1	1.59			
BMI	0.61	16.20	5.91 × 10^−5^*	1.18 × 10^−4^*
Systolic BP	0.35	12.18	0.0005*	7.20 × 10^−4^*
Diastolic BP	0.38	14.84	0.0001*	1.50 × 10^−4^*
APOE4*	−0.14	0.11	0.74	0.74

Abbreviations: *APOE4* = *APOE* ε4 carrier or noncarrier; BMI = body mass index; BP = blood pressure; *p*corr = false discovery rate–adjusted *p* values; TP = time point.

Participant sample size = 1,120 at baseline; n = 1,088 with *APOE* genotype available. Beta values represent the coefficients derived from the regression outputs (eTable 3 for full output). The reference category for sex is male. The reference category for *APOE4* is noncarriers.

*p* Values <0.05 are marked with *.

### Interaction of Sex, CMRs, and Time Point on BAG (Models 2–4)

Our analyses revealed significant 2-way interaction effects between sex and CMRs on BAG (model 2). Supplementary analyses further revealed significant interactions involving sex and *APOE4* status and sex and time point on BAG. eTable 4 in the Supplement presents ANOVA outputs investigating the 2-way interactions on BAG.

Our analyses assessing sex differences in the associations between BAG and CMRs by time point (model 3) revealed significant 3-way interaction effects for all 3 CMRs, as shown in Table 3 (eTable 5 for the regression outputs of model 3). As shown in eTable 6, within-sex post hoc analyses clarifying the interactions between CMRs and time point (model 4) showed significant interactions for all 3 CMRs in females and only for BMI in males. eTables 7–9 provide the regression outputs for each sex for model 4.

**Table 3:** Sex Differences in the Associations Between Brain Age Gap and BMI, SBP, DBP, and TP (Model 3)

Interaction	β	*F*	*p* Value	*p*corr
Sex × BMI × TP		6.55	7.65 × 10^−7^*	2.30 × 10^−6^*
Male × BMI × TP1	0.99			
Male × BMI × TP2	0.97			
Male × BMI × TP3	0.71			
Female × BMI × TP1	0.67			
Female × BMI × TP2	0.23			
Female × BMI × TP3	−0.35			
Sex × SBP × TP		3.14	0.005*	0.005*
Male × SBP × TP1	0.38			
Male × SBP × TP2	0.13			
Male × SBP × TP3	−0.02			
Female × SBP × TP1	0.62			
Female × SBP × TP2	0.32			
Female × SBP × TP3	0.50			
Sex × DBP × TP		3.79	0.0009*	0.001*
Male × DBP × TP1	0.52			
Male × DBP × TP2	0.27			
Male × DBP × TP3	0.21			
Female × DBP × TP1	0.67			
Female × DBP × TP2	0.32			
Female × DBP × TP3	0.07			

Abbreviations: BMI = body mass index; BP = blood pressure; DBP = diastolic BP; *p*corr = false discovery rate–adjusted *p* values; SBP = systolic BP; TP = time point.

Participant sample size = 1,120 at baseline. Beta values represent the coefficients derived from the regression outputs (eTable 5 for full output).

*p* Values <0.05 are marked with *.

**Figure 2:**
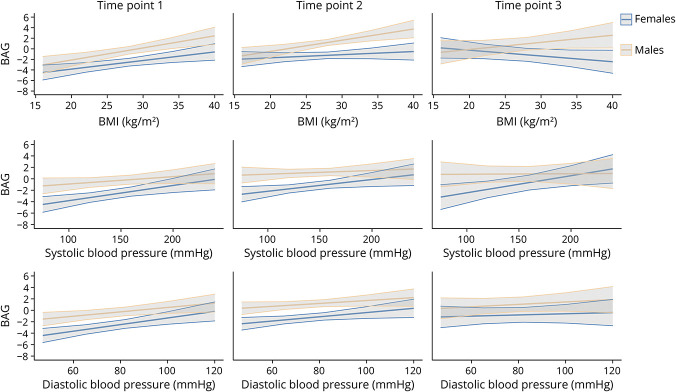
Relationships Between CMRs and BAG in females and males Fitted linear relationships between BAG and BMI, systolic BP, and diastolic BP in females and males, with their corresponding 95% CIs (model 3). BAG = brain age gap; BMI = body mass index; BP = blood pressure; CMR = cardiometabolic risk factor.

In females, higher BMI was associated with higher BAG at time point 1 (Figure 2, eTable 7). Post hoc pairwise comparisons indicated that the association between BMI and BAG was significantly stronger at time point 1 compared with subsequent time points, although these differences were small (eTable 10).

In males, BMI was positively associated with BAG at the first 2 time points (Figure 2, eTable 7). Post hoc pairwise comparisons revealed no significant differences in the association between BMI and BAG between time points (eTable 10).

In females, higher systolic and diastolic BP levels were associated with higher BAG (Figure 2, eTables 8 and 9 provide regression outputs for each group per time point). Post hoc pairwise comparisons revealed no significant differences in the association between systolic BP and BAG between time points (eTable 10). However, the association between diastolic BP and BAG was significantly stronger at time point 1 compared with time point 3, although these differences were small (eTable 10). In males, the 2-way interactions of the relationship between BAG and BP and time point were not statistically significant (eTable 6).

### Sex-Specific Interactions of CMR, Time Point, and *APOE4* Status on BAG (Model 5)

**Figure 3:**
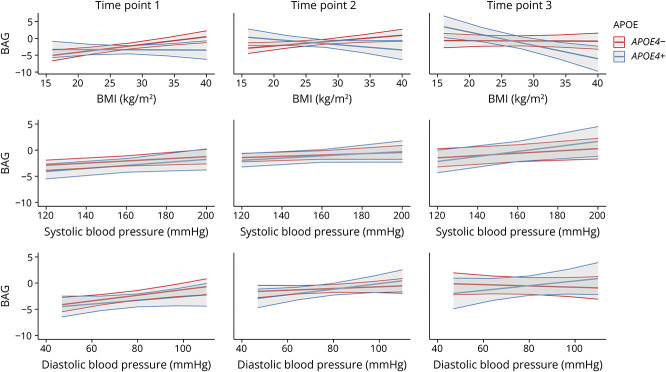
Interactions Between CMRs, *APOE4* Status, and Time Point on BAG in females The fitted interaction between BMI (top), systolic BP (middle), and diastolic BP (bottom); *APOE4* status; and time point on BAG in females, with their corresponding 95% CIs (model 4). BAG = brain age gap; BMI = body mass index; BP = blood pressure; CMR = cardiometabolic risk factor.

In females only, there was a significant interaction effect of *APOE4* status with time point across all 3 CMRs (Table 4, Figure 3). eTables 11–13 provide the regression outputs for each sex used in model 5.

**Table 4:** The Interaction Between Cardiometabolic Risk Factors (BMI, SBP, DBP), *APOE4* Status, and TP on Brain Age Gap in females and males Separately (Model 5)

Interaction	Female				Male			
	β	*F*	*p* Value	*p*corr	β	*F*	*p* Value	*p*corr
BMI × TP × *APOE4*		6.16	2.63 × 10^−6^*	7.89 × 10^−6^*		2.26	0.04*	0.06
BMI × TP1 × *APOE4*−	0.99				0.83			
BMI × TP2 × *APOE4*−	0.68				0.60			
BMI × TP3 × *APOE4*−	0.04				0.41			
BMI × TP1 × *APOE4*+	−0.13				0.31			
BMI × TP2 × *APOE4*+	−0.79				0.81			
BMI × TP3 × *APOE4*+	−1.75				0.99			
SBP × TP × *APOE4*		2.24	0.04*	0.04*		1.25	0.28	0.28
SBP × TP1 × *APOE4*−	0.50				0.26			
SBP × TP2 × *APOE4*−	0.15				0.32			
SBP × TP3 × *APOE4*−	0.27				−0.08			
SBP × TP1 × *APOE4*+	0.51				0.47			
SBP × TP2 × *APOE4*+	0.25				−0.34			
SBP × TP3 × *APOE4*+	0.93				0.01			
DBP × TP × *APOE4*		2.81	0.01*	0.02*		2.60	0.02*	0.06
DBP × TP1 × *APOE4*−	0.66				0.38			
DBP × TP2 × *APOE4*−	0.11				0.31			
DBP × TP3 × *APOE4*−	−0.18				0.52			
DBP × TP1 × *APOE4*+	0.34				1.13			
DBP × TP2 × *APOE4*+	0.39				0.43			
DBP × TP3 × *APOE4*+	0.40				−0.19			

Abbreviations: BMI = body mass index; BP = blood pressure; DBP = diastolic BP; *p*corr = false discovery rate-adjusted *p* values; SBP = systolic BP; TP = time point.

Participant sample size = 1,088 at baseline. Beta values represent the coefficients derived from the regression outputs (eTables 11–13 for full output).

*p* Values <0.05 are marked with *.

Higher BMI was associated with higher BAG in female noncarriers, but with lower BAG in female carriers, particularly at time point 3 (eTable 11). Post hoc pairwise comparisons indicated small but significant differences in associations between BAG and BMI as a function of *APOE4* status and time point (eTable 14).

A positive association between systolic/diastolic BP and BAG was observed across female carriers and noncarriers (Table 4, Figure 3, eTables 12 and 13). Post hoc pairwise comparisons revealed that in noncarriers, the association between diastolic BP and BAG was stronger at time point 1 compared with time point 3, although this difference was small. There were no other significant differences in these associations as a function of *APOE4* status or time point (eTables 15 and 16).

In males, the 3-way interactions examining the relationships between BAG and CMRs, *APOE4* status, and time point did not reach statistical significance (Table 4, eFigure 4).

Supplementary sensitivity and covariate analyses were consistent with our primary analyses (eMethods 1 and 2, eTables 17–74).

## Discussion

This longitudinal study leveraged over 1,100 cognitively normal older participants from 2 unique narrow-age cohorts, identifying sex differences in relationships between CMRs, *APOE* genotype, and BAG over time. Specifically, the results indicate stronger correlations between CMRs and BAG in females compared with males, with the associations between BMI and BAG further varying by *APOE4* status exclusively in females.

Previous cross-sectional studies^4,5,36^ align with our longitudinal study findings, confirming contrasting relationships between CMRs and brain health in females and males. Our findings clarify that these associations are not solely attributed to generational or cohort-related effects but also may stem from dynamic differences in how CMRs relate to brain health over time. In females, greater BMI was linked to higher BAG in *APOE4* noncarriers, but lower BAG in carriers, a difference that was more pronounced at the last observation compared with those at younger ages. Because participants at higher risk of AD may be more vulnerable to frailty or weight loss,^5,17^ higher BMI in female *APOE4* carriers may signal healthy fat/muscle and preserved brain health^37^ while lower BMI could indicate preclinical neurodegeneration.^17^ However, our results were consistent after excluding participants with a dementia diagnosis and MMSE scores indicative of cognitive impairments. Future studies should explore sex differences in associations between CMRs and brain health in cohorts including larger patient groups to clarify potential links to observed sex differences in dementia prevalence.^38^ Furthermore, body fat in postmenopausal females may serve as an endogenous source of estrogen, compensating for the menopause-related decline in estrogen production in the ovaries.^16^ Sex-specific metabolomic differences, influenced by body fat, may also affect brain health.^39^ For example, a recent study involving over 9,000 older adults showed that differences in circulating metabolites and lipid measures were associated with variations in white matter hyperintensity volume in males vs females.^40^

In males, greater BMI was associated with higher BAG across carriers and noncarriers, consistent with our previous UK Biobank study showing similar associations regardless of *APOE4* status.^4^ Although the brain-body relationships in males contrasted with the patterns observed in females, the associations observed in females were also independent of *APOE4* status in the UK Biobank cohort.^4^ Cohort characteristics, such as age, may influence *APOE4* status effects on brain age. One strength of the cohorts in this study is that participants were all born in the same year—1936 for LBC1936 and 1946 for Insight 46. The distinct advantage of such narrow-age cohorts, as showcased in our study, is the reduced potential for age-related confounding effects on the findings. Further research is required to clarify our findings, particularly regarding *APOE* dosage, such as comparing the effects in participants with 2 ε4 alleles with those with 1. Unfortunately, we were unable to conduct these analyses here because of the small subsamples within each *APOE* group.

In females specifically, higher BP was generally linked to higher BAG. These findings align with previous research demonstrating the detrimental impacts of hypertension on brain health in females.^41^ While we accounted for medication use in our supplementary analyses, data on the type of medication (e.g., cardiovascular-related vs other health-related) could further clarify the relationship between BP, *APOE4* status, and brain health in males and females over time. Furthermore, the potential influence of other unexplored CMRs, such as a history of coronary heart disease, may have a significant impact on brain structure.^42^ Future research could investigate a broader range of CMRs,^2,43^ exploring their complex interactions and potential nonlinear influences on brain health in males and females with age. Although we observed statistically significant differences in associations between CMRs and BAG between time points, the differences were small, likely due to the short time interval between time points in a cognitively normal cohort. This underscores the necessity for longitudinal cohorts with longer time frames in future studies.

While our focus was on participant sex, gender may further clarify the relationships between CMRs, *APOE4* status, and brain age over time.^44^ Gender-related factors, such as the increased caregiving responsibilities often shouldered by womales, might be linked to higher stress levels and increased risk of hypertension, compounded by other lifestyle and psychosocial challenges.^45^ Our samples predominantly consisted of White participants from the United Kingdom, restricting the generalizability of our results to more diverse cohorts. In addition, the cohorts displayed relatively high levels of educational achievements, suggesting that participants with varied socioeconomic backgrounds may have been underrepresented in this study.^46^ The limited diversity in our samples emphasizes the need for further exploration and research within more diverse cohorts.^44^ Future work should also consider sex-related and gender-related group differences in rates of attrition. Approximately 20% of participants dropped out between time points in each cohort, with previous studies indicating characteristics such as lower cognition, socioeconomic status, and physical fitness as key reasons for dropout.^21,47^ While these attrition rates are comparable with those of other longitudinal aging studies,^48,49^ investigating potential sex-specific biases in participant retention might further clarify observed differences in cardiometabolic risk and impacts on brain health over time.

In summary, this study highlights sex differences in the associations between CMRs and *APOE4* status on brain age, emphasizing the importance of conducting sex-stratified analyses to examine aging trajectories in females and males. All 3 CMRs showed associations with brain health in females while BMI was the primary CMR relating to brain health in males. The associations between BMI and BAG further depended on *APOE4* status exclusively in females. These sex-specific patterns in the association between CMRs and brain health may contribute to divergent aging trajectories and neurodegenerative disease risks. Notably, studies focusing on sex differences in cardiometabolic risk and brain health are largely lacking. Given the increasing prevalence of older adult demographics and the higher risk of neurodegenerative diseases with sex-specific etiologies,^50^ our findings provide further motivation to clarify the sex-specific associations between cardiometabolic and brain health over the adult lifespan.

## Appendix Authors

**Table:**

Name	Location	Contribution
Sivaniya Subramaniapillai, PhD	Department of Clinical Neuroscience, Lausanne University Hospital and University of Lausanne, Switzerland	Drafting/revision of the manuscript for content, including medical writing for content; study concept or design; analysis or interpretation of data
Louise S. Schindler, MSc	Department of Clinical Neuroscience, Lausanne University Hospital and University of Lausanne, Switzerland	Drafting/revision of the manuscript for content, including medical writing for content; analysis or interpretation of data
Paul Redmond, MSc	Department of Psychology, University of Edinburgh, United Kingdom	Drafting/revision of the manuscript for content, including medical writing for content; major role in the acquisition of data
Mark E. Bastin, DPhil	Department of Psychology, University of Edinburgh, United Kingdom	Drafting/revision of the manuscript for content, including medical writing for content
Joanna M. Wardlaw, MD, FRCR	Department of Psychology, University of Edinburgh, United Kingdom	Drafting/revision of the manuscript for content, including medical writing for content; major role in the acquisition of data
Maria Valdés Hernández, PhD	Department of Psychology, University of Edinburgh, United Kingdom	Drafting/revision of the manuscript for content, including medical writing for content
Susana Muñoz Maniega, PhD	Department of Psychology, University of Edinburgh, United Kingdom	Drafting/revision of the manuscript for content, including medical writing for content
Benjamin Aribisala, PhD	Department of Psychology, University of Edinburgh, United Kingdom	Drafting/revision of the manuscript for content, including medical writing for content
Lars T. Westlye, PhD	Department of Psychology, University of Oslo, Norway	Drafting/revision of the manuscript for content, including medical writing for content; analysis or interpretation of data
William Coath, MSc	Dementia Research Centre, University College London, United Kingdom	Drafting/revision of the manuscript for content, including medical writing for content; major role in the acquisition of data
James Groves, MBBS	Dementia Research Centre, University College London, United Kingdom	Drafting/revision of the manuscript for content, including medical writing for content; major role in the acquisition of data
David M. Cash, PhD	Dementia Research Centre, University College London, United Kingdom	Drafting/revision of the manuscript for content, including medical writing for content; major role in the acquisition of data
Josephine Barnes, PhD	Dementia Research Centre, University College London, United Kingdom	Drafting/revision of the manuscript for content, including medical writing for content
Sarah-Naomi James, PhD	Dementia Research Centre, University College London, United Kingdom	Drafting/revision of the manuscript for content, including medical writing for content
Carole H. Sudre, PhD	Dementia Research Centre, University College London, United Kingdom	Drafting/revision of the manuscript for content, including medical writing for content
Frederik Barkhof, MD, PhD	Centre for Medical Image Computing, University College London, United Kingdom	Drafting/revision of the manuscript for content, including medical writing for content; major role in the acquisition of data
Marcus Richards, PhD	MRC Unit for Lifelong Health and Ageing, University College London, United Kingdom	Drafting/revision of the manuscript for content, including medical writing for content; major role in the acquisition of data
Janie Corley, PhD	Department of Psychology, University of Edinburgh, United Kingdom	Drafting/revision of the manuscript for content, including medical writing for content
Tom C. Russ, MD, PhD	Department of Psychology, University of Edinburgh, United Kingdom	Drafting/revision of the manuscript for content, including medical writing for content
Simon R. Cox, PhD	Department of Psychology, University of Edinburgh, United Kingdom	Drafting/revision of the manuscript for content, including medical writing for content; major role in the acquisition of data
Jonathan M. Schott, MD	Dementia Research Centre, University College London, United Kingdom	Drafting/revision of the manuscript for content, including medical writing for content; major role in the acquisition of data
James H. Cole, PhD	Centre for Medical Image Computing, University College London, United Kingdom	Drafting/revision of the manuscript for content, including medical writing for content; study concept or design; analysis or interpretation of data
Ann-Marie G. de Lange, PhD	Department of Clinical Neuroscience, Lausanne University Hospital and University of Lausanne, Switzerland	Drafting/revision of the manuscript for content, including medical writing for content; study concept or design; analysis or interpretation of data

## Glossary

AD: Alzheimer disease
BAG: brain age gap
BF%: body fat percentage
BMI: body mass index
BP: blood pressure
CMR: cardiometabolic risk factor
LBC1936: Lothian Birth Cohort 1936
lmer: linear mixed-effects regressions
WHR: waist-to-hip ratio
